# Epigenetic modulation of antitumor immunity and immunotherapy response in breast cancer: biological mechanisms and clinical implications

**DOI:** 10.3389/fimmu.2023.1325615

**Published:** 2024-01-10

**Authors:** Jun Yin, Tiezheng Gu, Norin Chaudhry, Nancy E. Davidson, Yi Huang

**Affiliations:** ^1^ The University of Pittsburgh Medical Center (UPMC) Hillman Cancer Center, School of Medicine, University of Pittsburgh, Pittsburgh, PA, United States; ^2^ Department of Internal Medicine, Division of Hematology, Oncology, and Blood and Marrow Transplantation, Carver College of Medicine, University of Iowa, Iowa City, IA, United States; ^3^ Fred Hutchinson Cancer Center, University of Washington, Seattle, WA, United States; ^4^ Holden Comprehensive Cancer Center, University of Iowa, Iowa City, IA, United States

**Keywords:** breast cancer, epigenetic alterations, DNA methylation, histone modifications, non-coding RNA, epigenetic therapy, immunotherapy, combination therapy

## Abstract

Breast cancer (BC) is the most common non-skin cancer and the second leading cause of cancer death in American women. The initiation and progression of BC can proceed through the accumulation of genetic and epigenetic changes that allow transformed cells to escape the normal cell cycle checkpoint control. Unlike nucleotide mutations, epigenetic changes such as DNA methylation, histone posttranslational modifications (PTMs), nucleosome remodeling and non-coding RNAs are generally reversible and therefore potentially responsive to pharmacological intervention. Epigenetic dysregulations are critical mechanisms for impaired antitumor immunity, evasion of immune surveillance, and resistance to immunotherapy. Compared to highly immunogenic tumor types, such as melanoma or lung cancer, breast cancer has been viewed as an immunologically quiescent tumor which displays a relatively low population of tumor-infiltrating lymphocytes (TIL), low tumor mutational burden (TMB) and modest response rates to immune checkpoint inhibitors (ICI). Emerging evidence suggests that agents targeting aberrant epigenetic modifiers may augment host antitumor immunity in BC via several interrelated mechanisms such as enhancing tumor antigen presentation, activation of cytotoxic T cells, inhibition of immunosuppressive cells, boosting response to ICI, and induction of immunogenic cell death (ICD). These discoveries have established a highly promising basis for using combinatorial approaches of epigenetic drugs with immunotherapy as an innovative paradigm to improve outcomes of BC patients. In this review, we summarize the current understanding of how epigenetic processes regulate immune cell function and antitumor immunogenicity in the context of the breast tumor microenvironment. Moreover, we discuss the therapeutic potential and latest clinical trials of the combination of immune checkpoint blockers with epigenetic agents in breast cancer.

## 1 Introduction

**Figure 1:**
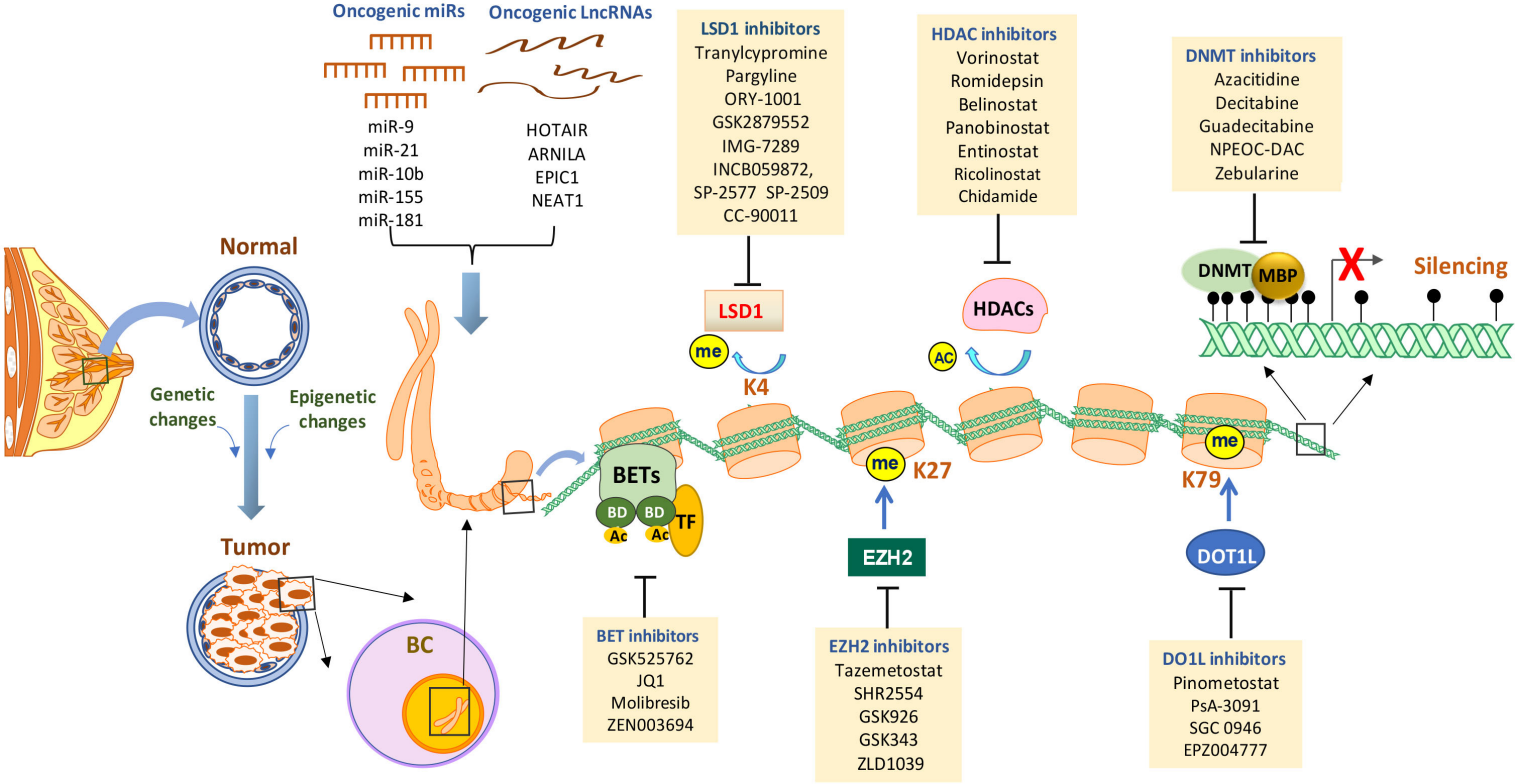
Targeting epigenetic alterations in breast cancer. Epigenetic alterations are critical drivers of breast tumor initiation and progression. The primary epigenetic changes in breast cancer include promoter DNA hypermethylation (black lollipops - methylated CpG dinucleotides), histone posttranslational modifications (enhanced histone deacetylation, aberrant histone methylation, BET amplification, etc), dysregulated expression and activity of miRNAs and lncRNAs. At a gene promoter, DNA methylation can be induced along with abnormal histone modifications and/or other epigenetic alterations that may result in the abnormal loss of genes with important functions in curbing breast tumor. Established or investigational drugs that can inhibit the activity of specific epigenetic modifiers are shown in the boxes. BC, breast cancer; DNMT, DNA methyltransferase; MBD, methyl-binding domain protein; HDAC, histone deacetylase; LSD1, lysine-specific demethylase 1; EZH2, enhancer of zeste homolog 2; DOT1L, DOT1-like histone lysine methyltransferase; BET, bromodomain and extra-terminal; miR, micro-RNA; lncRNA, long non-coding RNA; TF, transcription factors; Ac, acetylation; Me, methylation.

Breast cancer (BC) is still a major threat and challenge for women’s health worldwide. Like many other types of cancer, breast cancer progression involves multiple steps of uncontrolled cell proliferation and aberrant apoptosis through gene expression changes including gain-of-function of oncogenes or loss-of-function of tumor suppressor genes (TSGs). The loss of TSGs can result from specific DNA mutations, deletion, or frame shifts. Another general mechanism by which normally expressed genes can be changed is so called “epigenetic modifications” (1–3). Three primarily interconnected epigenetic mechanisms have been identified: DNA methylation, post-translational modifications (PTM) of histones, and non-coding RNAs (ncRNA) (4, 5). Several epigenetic-based drugs (DNMT, HDAC and EZH2 inhibitors) have been approved by the FDA for clinical treatment of hematological and solid malignancies (**Table 1**). In breast cancer, epigenetic alterations drive breast tumor proliferation, invasion and metastasis through genome-wide loss and local gains of DNA methylation within promoters of TSGs, disorders in histone modifications, and abnormal expression of noncoding RNAs (6–9). There is a considerable interest in the potential of epigenetic dysregulation as biomarkers and therapeutic targets in BC. The association between epigenetic aging and clinical outcomes has been recently evaluated in longer term BC survivors to guide cancer care for older women (10). Epigenetic malfunctions can potentially be restored to their normal phenotypes through epigenetic-based therapies (**Figure 1**). A number of promising epigenetic modifying drugs are under clinical investigation at various stages of breast cancer treatment as a single agent or administered in combination with other therapeutic drugs (11, 12) (**Table 2**).

**Table 1:** Epigenetic drugs approved by the FDA for treatment of cancer.

Drug	Epigenetic Target	Clinical Applications	Year of Approval	Approval Agent
Azacitidine	DNMT	Myelodysplastic syndrome (MDS)	2004	US FDA
Decitabine	DNMT	Myelodysplastic syndrome (MDS)	2006	US FDA
Vorinostat	HDAC	Cutaneous T-cell lymphoma (CTCL)	2006	US FDA
Romidepsin	HDAC	Cutaneous T-cell lymphoma (CTCL)	2009	US FDA
Belinostat	HDAC	Peripheral T-cell lymphoma (PTCL)	2014	US FDA
Panobinostat	HDAC	Multiple myeloma	2015	US FDA
Chidamide	HDAC	Relapsed or refractory (R/R) peripheral T cell lymphoma (PTCL)	2015	China FDA
Tazemetostat	EZH2	Epithelioid sarcoma	2020	US FDA

**Table 2:** Current stages of leading epigenetic drugs for breast cancer therapy.

Drug targets	Compounds	Study stage	Ongoing clinical trials in breast cancer
**DNMT inhibitor**	Azacitidine Decitabine Guadecitabine NPEOC-DAC	Phase II Phase II Preclinical Preclinical	NCT04891068, NCT01349959, NCT05381038, NCT02957968, NCT05673200
**HDAC inhibitor**	Vorinostat Romidepsin Panobinostat Belinostat Entinostat Ricolinostat Citarinostat KA2507	Phase II Phase II Phase II Phase I Phase III Phase I Preclinical Preclinical	NCT03742245, NCT00416130, NCT00616967 NCT02393794, NCT01638533 NCT03878524 NCT04315233, NCT04703920 NCT01349959, NCT03538171, NCT02115282, NCT02453620, NCT03280563
**LSD1 inhibitor**	Tranylcypromine Phenelzine sulfate ORY-1001 GSK2879552 INCB059872 HCI-2577	Preclinical Phase I Preclinical Preclinical Preclinical Preclinical	
**EZH2 inhibitor**	Tazemetostat SHR2554 GSK926 GSK343 ZLD1039	Preclinical Phase II Preclinical Preclinical Preclinical	NCT04355858
**BET inhibitor**	JQ1 Molibresib ZEN003694	Preclinical Phase II Phase II	NCT03901469, NCT05422794
**DOT1L inhibitor**	Pinometostat PsA-3091 SGC 0946 EPZ004777	Preclinical Preclinical Preclinical Preclinical	

Immunotherapy has opened a new era in cancer treatment with the FDA approval of several immune checkpoint inhibitors (ICI) which block checkpoint proteins from binding with their partner proteins (e.g. PD-1, PD-L1, CTLA-4, LAG-3) in tumors (13, 14). The immune system plays a critical role in surveillance against the initiation and development of breast cancer by recognizing tumor associated antigen (TAA) and eliciting immunogenic cell death (ICD) (15). Although breast cancer has been traditionally viewed to be immunologically silent, treatment with ICIs has been shown to improve clinical outcomes in some patients with metastatic BC, especially triple negative breast cancer (TNBC) (16, 17). In 2021, Pembrolizumab (anti-PD-1 mAb) in combination with chemotherapy was approved by FDA as a neoadjuvant/adjuvant treatment for high-risk, early-stage TNBCs (18). However, only a small fraction of BC patients can benefit from immunotherapy, and the overall response rates of BC to immunotherapy were only 10-20% (19, 20). Therefore, there is an urgent necessity for the development of more effective clinical strategies for BC patients whose tumors are less immunogenic and refractory to immunotherapy.

Emerging evidence has highlighted key roles of epigenetic alterations in impaired anti-tumor immunity, immune escape, and immunotherapy resistance in breast tumors. Epigenetic regulation influences all aspects of the interaction between BC cells and immune system, thus providing a rational basis for efforts to convert a breast tumor from an immune suppressive (cold) to an immune permissive (hot) state through joint epigenetic therapy. The combination of epigenetically targeted drugs and ICIs is being investigated in clinical trials in a variety of cancer types including BC. In this review, we highlight the evidence of the new roles of epigenetic crosstalk between breast tumors and the immune environment. We also discuss the latest findings on epigenetic biomarkers for immunotherapy, emphasizing the current strategies to improve immunotherapy through reprogramming the breast cancer epigenome. Finally, we summarize the ongoing clinical trials that are evaluating the combination of immune checkpoint blockade with epigenetic agents against breast cancer.

## 2 Epigenetic biomarkers and targets in breast cancer

### 2.1 DNA methylation

DNA methylation is catalyzed by a group of enzymes termed DNA methyltransferases (DNMTs) which covalently modify the C-5 position of cytosine residues in CpG islands, using S-adenosylmethionine as a methyl donor. DNMTs are divided into maintenance (DNMT1) and *de novo* methyltransferases (DNMT3a, DNMT3b) (21, 22). Many cancer-related genes have been shown to be hypermethylated in BC cells. These genes have a wide range of cellular functions that are involved in regulation of hormone activity (e.g. *ERα*, *PR)*, cell cycle and apoptosis (e.g. *RARb*, *p16*, *CCND2, SFRPs*, *RASSF1a*, *TMS1, APC*), DNA damage repair (e.g. *BRCA1*, *14-3-3σ*, *HIC1*, *MLH1*), invasion and metastasis (e.g. *E-cadherin*, *TIMP-3*) (3, 6, 7, 23, 24). A recent AURORA US Metastasis Project analyzed patients with metastatic BC by RNA sequencing, exome and whole-genome sequencing and global DNA methylation microarrays and revealed that DNA methylation downregulates ER-mediated cell-cell adhesion genes in metastases (25). Thus, DNA methylation represents a novel opportunity to identify biomarkers for early screening and diagnosis, prognosis, outcome prediction, and treatment monitoring of BC (26, 27).

Blockade of DNMT activity is the most effective approach to inhibit DNA methylation. Two DNMT-inhibiting cytosine nucleoside analogues, 5-aza-cytidine (azacitidine) and 5-aza-2’-deoxycytidine (decitabine), received FDA approval for the treatment of hematologic cancers. These two drugs induce antineoplastic activity by inhibition of DNMT activity at low dose and direct cytotoxicity at high dose through incorporation into newly synthesized DNA strands as nucleoside analogs to disrupt normal DNA synthesis. Recently, some new DNMT inhibitors (DNMTi) have been identified, such as guadecitabine (SGI-110), zebularine, and NPEOC-DAC, which exhibit increased resistance to degradation from deamination by cytidine deaminase and prolong plasma half-life (28, 29). Preclinical studies have demonstrated the effectiveness of DNMTi in restoring aberrantly silenced genes and reprograming the epigenome that may block BC cell proliferation and/or sensitize tumors to other therapeutic interventions (30–32). However, early clinical trials showed that DNMTi has limited efficacy as monotherapy in clinical studies in BC, in part due to their toxicity and off-target effects. Current clinical trials are exploring novel strategies through combining DNMTi with other therapies aiming to maximize therapeutic efficacy and minimize side effects. The combination of DNMTi and chemotherapy has been intensively studied in BC preclinical studies and showed enhanced susceptibility compared to chemotherapy alone (33–35). The safety and efficacy of combinations of DNMTi and standard chemotherapeutics are currently being assessed in multiple breast cancer clinical trials.

### 2.2 Histone acetylation and deacetylation

Histone acetyltransferases (HATs) and histone deacetylases (HDACs) are the enzymes responsible for catalyzing the addition or removal of acetyl groups respectively. Aberrant overexpression of HDACs correlates with aggressive clinicopathological features and poor prognosis of BC (36, 37). The development of HDAC inhibitors (HDACi) has produced encouraging results in the clinic, particularly in the field of hematological malignancy. HDACi are powerful epigenetic modulators that may exert antineoplastic effects by re-expressing silenced TSGs, inducing cancer cell cycle arrest and apoptosis, reducing angiogenesis, and modulating immune response (7, 38). In ER-negative BC cells, overexpression of HDACs have been shown to suppress transcriptional activity of key hormonal receptors, ERα and PR, and treatment with HDACi led to functional reactivation of these silenced receptors either alone or in combination with DNMTi (30, 39, 40). Inhibition of SIRT1, a member of class III HDAC, has been shown to restore multiple epigenetically suppressed TSGs, such as *SFRP1*, *SFRP2*, *E*-*cadherin* and *CRBP1* in BC (41). Thus far, four pan-HDACi, vorinostat (Zolinza), romidepsin (Istodax), panobinostat (Farydak), and belinostat (Beleodaq), have been approved by the FDA for the treatment of hematological malignancies. However, clinical studies found that use of HDACi in solid tumors, including BC, showed limited single-agent activity (42, 43). An early window trial observed limited effects of orally administered vorinostat on chromatin marks and methylated genes in newly diagnosed early-stage BC patients although the treatment led to significant reduction in expression of proliferation-related genes (*Ki-67*, *STK15*, *Cyclin B1*) (42). Another early phase trial of vorinostat in metastatic BC patients did not meet the RECIST response criteria for adequate single-agent activity (43). Several clinical trials are evaluating the efficacy of combining HDACi with other standard cancer therapies (e.g. aromatase inhibitor, chemotherapy, PARP inhibitors, CDK4/6 inhibitors, anti-HER2 therapy) in breast cancer. One recent phase III trial reported that HDAC inhibitors exemestane and entinostat did not improve survival in patients with aromatase inhibitor-resistant advanced BC although pharmacodynamic analysis confirmed target inhibition of HDACi (44). It remains to be determined if there is a role for HDACi in a biomarker-selected BC patient population based on results from ongoing correlative clinical trials. These clinical data suggest that therapeutic potential of current HDACi, although effective in hematological malignancies, is frequently affected by off-target effect, low specificity, unfavorable pharmacokinetics with a relatively short half-life and fast clearance in solid tumor context. These challenges highlight the need for development of the next-generation HDACi with optimized activity and selectivity profiles.

### 2.3 Histone methylation and demethylation

Histone methylation occurs predominantly on lysine (K) and arginine (R) residues on the H3 and H4 tails which is dynamically regulated by specific lysine methyltransferases (KMT) and demethylases (KDM). There is mounting and compelling evidence that dysregulation of KMTs or KDMs leads to altered histone methylation patterns, which may contribute to BC pathogenesis (6, 7, 45). Enhancer of Zeste Homolog 2 (*EZH2*), a subunit of Polycomb Repressive Complex 2 (*PRC2*), catalyzes the addition of methyl groups to histone H3 at lysine 27 (H3K27me), which is typically associated with repression of genes expression (46). EZH2 is often elevated in breast carcinoma and excessive EZH2 expression facilitates invasive tumor growth and aggressive clinical behavior (47, 48). A number of EZH2 inhibitors have been evaluated for their anti-cancer properties, and many clinical trials are under way to examine the antitumor efficacy of EZH2i alone or in combination with other anticancer drugs. In 2020, tazemetostat (Tazverik), a first-in-class small molecule EZH2 inhibitor, was granted accelerated approval by the FDA for patients with advanced or metastatic epithelioid sarcoma who are not eligible for curative surgery. Being the first approval of an epigenetic drug for a solid tumor, tazemetostat represents a new milestone in the cancer epigenetic field.

The histone methyltransferase responsible for H3K79 methylation is an enzyme named DOT1L (disruptor of telomeric silencing 1 like) that is a non-SET domain-containing methyltransferase involved in gene transcription, heterochromatin formation, and DNA repair (49). Oktyabri et al. reported that DOT1L and its target gene, *BCAT1* (branched-chain amino acid transaminase) were both up-regulated in BC cells which promotes sphere formation and cell migration in BC cells (50). A recent study showed that inhibition of DOT1L silences ERα gene and blocks the proliferation of antiestrogen-resistant BC cells, indicating that DOT1L is an attractive epigenetic target for treating endocrine therapy-resistant ER+ BC (51). Inhibition of DOT1L and H3K79 methylation has been shown to selectively block proliferation, self-renewal, and metastatic properties in BC cells (52, 53). Byun et al. found that DOT1L is associated with high metastatic potential mediated by the EMT process in TNBCs, and treatment with a novel DOT1L inhibitor, psammaplin A analog (PsA-3091), induced E-cadherin and hindered TNBC progression both *in vitro* and *in vivo* (53). These studies suggest that the use of DOT1L inhibitors is emerging as a new therapeutic approach for aggressive BC.

Histone lysine-specific demethylase 1 (LSD1, AOF2, or KDM1A) is the first discovered FAD-dependent histone demethylase that is typically associated with a transcriptional repressor complex such as CoREST, HDAC1/2, BHC80, etc (54–56). The canonical function of LSD1 is the demethylation of lysine 4 of histone 3 (H3K4me), leading to chromatin inaccessibility and gene transcription silencing (54, 55, 57, 58). LSD1 expression is markedly elevated during breast tumor progression from pre-invasive ductal carcinomas *in situ* (DCIS) to invasive ductal carcinoma (IDC) (59, 60). Analysis of TCGA data indicates a markedly elevated LSD1 expression in the TNBC/basal-like subtype in comparison to other BC groups (61). LSD1 overexpression is significantly associated with worse clinical outcomes in TNBC patients (60, 62). LSD1 has become an important validated epigenetic target for cancer therapy, and numerous small molecule inhibitors have been identified. Several leading LSD1 inhibitors, such as tranylcypromine, ORY-1001, IMG-7289, GSK2879552, INCB059872, CC90011 and SP-2577 (Seclidemstat), have advanced into clinical trials for neoplastic and other diseases.

LSD2 (KDM1B or AOF1) has been identified as a second member of the FAD-dependent histone demethylase family (63). LSD1 and LSD2 share significant similarities in the AO (amine oxidase) catalytic domain and FAD-dependent demethylation activity, but these two enzymes also display different properties such as patterns of chromatin complexes, transcriptional repression mechanisms, and genomic loci (64). We have shown that LSD2 is overexpressed in TNBC cells, which contributes to tumor proliferation and confers CSC-like characteristics (65). We also found that LSD2 overexpression induced global DNA methylation in TNBC. Inhibition of LSD2 reexpressed methylated TSGs and enhanced DNMTi-mediated apoptosis (66). Since LSD2 is part of chromatin-remodeling complexes distinct from those involving LSD1, further study is needed to elucidate the various roles of LSD2 in cell-context-specific settings.

Since the discovery of LSD1, many new KDMs have been identified and characterized. Most of these newly identified HDMs are members of the Jumonji family, which catalyze demethylation of histone lysine residues through a hydroxylation reaction using iron Fe^2+^ and α-ketoglutarate as cofactors (67–69). Xie et al. have demonstrated that KDM6A (UTX), a key demethylase of H3K27 mark, promotes hormone-responsive breast carcinogenesis through feed-forward transcriptional regulation with ER (70). KDM5B (PLU-1), a JmjC demethylase targeting H3K4me3, is implicated in the proliferative capacity of BC cells through direct transcriptional repression of TSGs including *BRCA1* (71). Overexpression of KDM4A was found in about 60% of BC tissue, and several KDM4A inhibitors have been investigated as anticancer agents in BC cells (72). Benedetti et al. reported that dual-KDM inhibitor (MC3324) targeting LSD1 and UTX induces growth arrest and apoptosis in hormone-responsive BC that is associated with a robust increase in H3K4me2 and H3K27me3 (73). These findings suggest that inhibition of FAD-dependent LSD1 or JmjC-based KDMs may represent a novel approach for BC therapy. Compared with the progress of LSD1 inhibitors, the development of agents targeting Jumonji KDMs is still in preclinical stage. The big challenge is that over 30 Jumonji KDMs have been identified, many with overlapping structures and functions. Thus, targeting a single KDM may have limited effect. A better understanding of the chromatin-context specificity of KDMs would help develop more effective and selective KDM inhibitors.

### 2.4 Bromodomain and extra-terminal family

The bromodomain and extraterminal domain (BET) family is comprised of four conserved mammalian members BRD2, BRD3, BRD4, and BRDT, which contain two similar tandem N-terminal bromodomains (BD1 and BD2). BET proteins are epigenetic readers that regulate gene transcription via binding to acetylated lysine residues on histone proteins and other nuclear factors (74, 75). BET proteins are frequently upregulated in BC cells leading to the activation of genes involved in tumor proliferation and metastasis (76–78). An increasing number of selective and pan-BET inhibitors (BETi) have been developed such as JQ1, I-BET151, I-BET762, OTX-015, ABBV-075, TEN-010, CPI-203, CPI-0610, etc. Li et al. showed that use of hypoxia-cleavable, RGD peptide-modified poly(D,L-lactide-co-glycolide) (PLGA) nanoparticle significantly improved delivery of JQ1 and therapeutic efficacy in suppressing primary breast tumors and bone metastasis (79). Multiple Phase I/II clinical trials have tested the safety and efficacy of BETi in solid tumors, including BC. While BET inhibition has shown promising preclinical anticancer activity in BC, some early clinical studies have reported dose limiting toxicity of BETi, including thrombocytopenia and gastrointestinal disorders, indicating a need for the development of well tolerated BETi and/or optimization of dosing schemes and combinations (76).

### 2.5 miRNA and lncRNA

MicroRNAs (miRNA) are endogenous non-coding single-stranded RNAs, 21-23-nucleotide in length, known for their pivotal roles in post-transcriptional modification and RNA silencing (80). A growing body of work has suggested that miRNAs play important roles in BC initiation and development. Overexpression of some oncogenic miRNAs (oncomiR), such as miR-9, miR-21, miR-29a, miR-155, miR-181, and miR-10b, etc, is correlated with advanced clinical stage, lymph node metastasis, and poor clinical outcome of BC (81–85). On the other hand, some miRNAs (let-7 family, miR-7, miR-145, miR-200 family, miR-205, miR-335, miR-30a, etc.) function as tumor suppressors to prevent breast cancer development through downregulating oncoproteins coding gene expression (86–89). miRNA-based therapies in BC aim to either inhibit oncomiRs via strategies such as antisense and oligonucleotides or induce tumor-suppressing miRNAs through epigenetic interventions (90, 91). In addition, chemically synthesized miRNA mimics have been used as novel classes of therapeutic agents to restore aberrantly reduced tumor-suppressing miRNAs to their normal levels (92).

Long non-coding RNAs (lncRNA) have been arbitrarily defined as non-protein-coding transcripts longer than 200 nucleotides that lack defined protein coding potential. Abnormal expression and activity of oncogenic lncRNAs, such as HOTAIR, ARNILA, EPIC1, NEAT1, and Lnc015192, accelerates BC proliferation and progression (93–98). HOTAIR is transcriptionally induced by TGFβ in BC cells and is required for EMT and stemness acquisition (99). By multivariate analysis of clinical samples from BC patients receiving neoadjuvant chemotherapy, Xu et al. showed that expression of an oncogenic lncRNA, EPIC1, was associated with ER negativity, HER-2 positivity, higher Ki67 expression, and higher histologic grade (100). These findings suggest that lncRNAs have the potential to serve as prognostic biomarkers and novel therapeutic targets for BC. Currently, several ongoing clinical trials are investigating the role of circulating lncRNAs as potential biomarkers for predicting the risk of recurrence in breast cancer.

## 3 Impact of epigenetics on cancer-immunity cycle and immune environment in breast cancer

**Figure 2:**
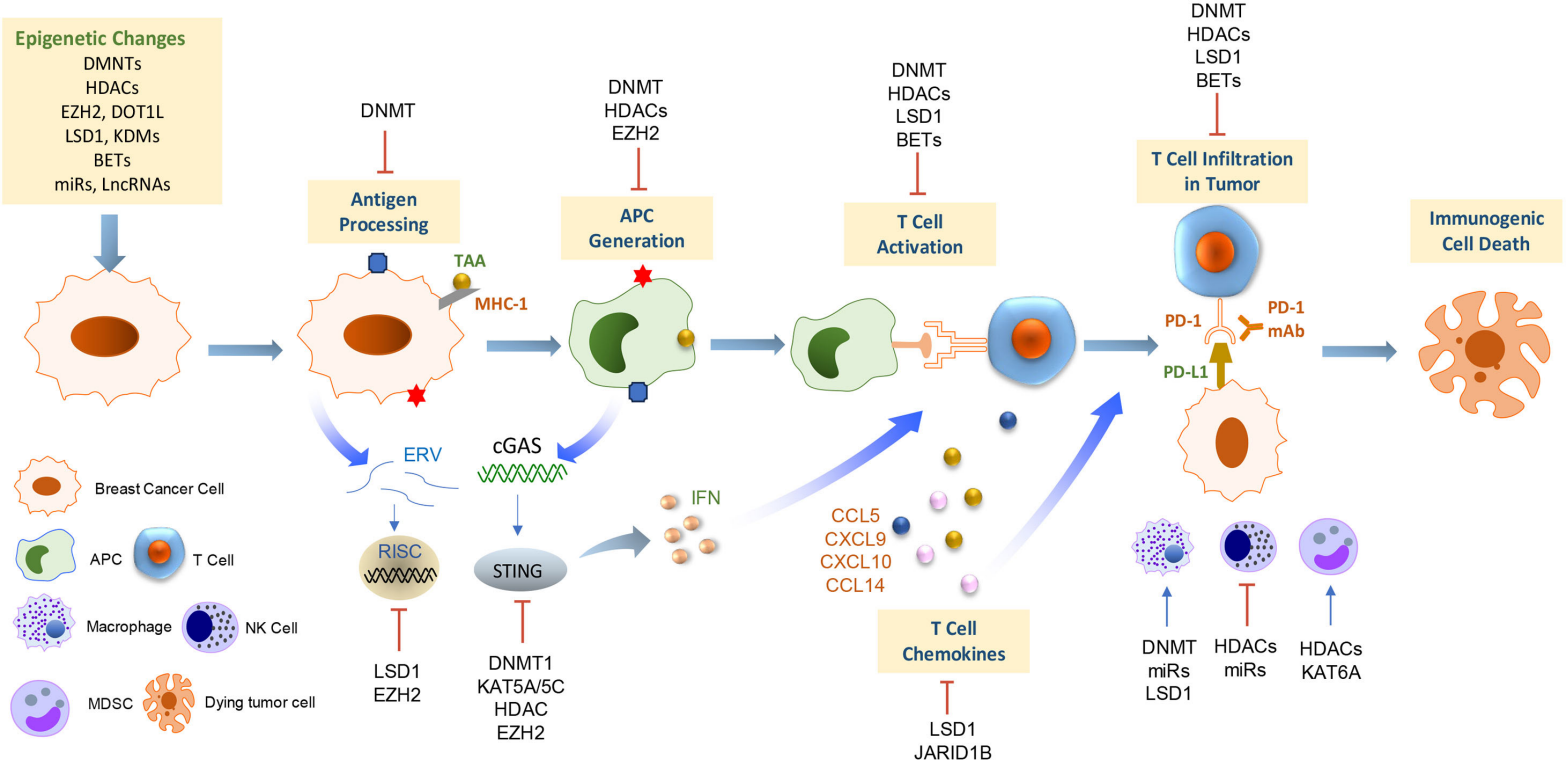
Epigenetic regulation of antitumor immunity and response to immunotherapy in breast cancer. This scheme summarizes the current understanding of the mechanisms underlying the epigenetic regulations on immune cell functions and antitumor immunogenicity in the context of the breast tumor microenvironment. APC, antigen-presenting cells; MDSC, myeloid-derived suppressor cell; NK cells, natural killer cells; TAA, tumor-associated antigen; ERV, endogenous retroviruses; cGAS, cyclic GMP–AMP synthase; STING, stimulator of interferon genes; IFN, interferon.

The normal mammary gland ductal layer contains different subsets of immune cells including lymphocytes (B cells and T cells), neutrophils, eosinophils, basophils, mast cells, monocytes, macrophages, dendritic cells, natural killer (NK) cells (101, 102). These immune cells are recruited to distinctive sites during the various stages of mammary gland development where they provide pathogen-specific, long-lasting protection to the breast epithelial layer and help eliminate transformed cells (103). Epigenetic modulations fine-tune gene expression patterns to maintain appropriate differentiation and function of immune cells in a homeostatic environment (104, 105). Growing evidence suggests that epigenetic defects in BC impair immune cell function and antitumor immunity, leading to a more immunosuppressive environment and tumor escape from immune surveillance (106, 107). In this section, we discuss the functional interaction between breast tumor cells and immune cells and how breast tumor hijacks epigenetic mechanisms to escape immune restriction and elimination (**Figure 2**).

In 2013, Chen and Mellman described the cancer-immunity cycle model of a series of self-sustaining stepwise events by which adaptive immune responses lead to effective elimination of tumor cells (108). Epigenetic regulations are pivotal factors for many processes in the cancer-immunity cycle (109). A variety of tumor-associated antigens (TAA) that are frequently expressed in BC cells can elicit strong antitumor immune responses. For example, cancer-testis antigens (CTAs) are a group of TAAs whose expression is restricted to malignant cells as well as some germline cells, thereby representing highly promising therapeutic targets for cancer immunotherapy because of their high tumor-specificity pattern and immunogenic nature (110, 111). VCX2, a member of the VCX/Y cancer/testis antigen family, is frequently repressed by promoter CpG methylation. Treatment with the DNMTi, guadecitabine, led to reactivation of VCX2 in several BC cell lines and a patient-derived xenograft (PDX) (112). Antigen presentation by major histocompatibility complex (MHC) is a critical response for adaptive antitumor immunity. MHC class I (MHC-I) molecules present peptide fragments derived from intracellular proteins and transport them to the cell surface (113). MHC-I loss or downregulation has been described as a major escape mechanism for tumors to evade immune surveillance and immunotherapy (114, 115). MHC-I genes are often methylated in human breast cancers, and treatment with guadecitabine can upregulate MHC-I expression in response to interferon γ that potentiates CD8+ T cell activity (116). In a high-throughput library screen of 141 epigenetic compounds, GSK-LSD1 (LSD1i), CUDC-101(inhibitor of HDAC, EGFR, and HER2) and BML-210 (HDACi) displayed significant antitumor effects and up-regulated MHC-I-mediated antigen presentation in orthotopic mammary gland tumors in mice (117). Recent research has explored the function and roles of endogenous retroviruses (ERV) as an additional source of tumor antigens (118). Deblois et al. showed that EZH2 suppressed ERV expression through H3K27me3 deposition, which prevented activation of the viral mimicry response and eluded the immune surveillance in chemotherapy-resistant BC (119). Induction of ERVs by LSD1 inhibition resulted in double-stranded RNA (dsRNA) stress and activation of type 1 IFN, leading to enhanced anti-tumor T cell immunity against growth of BC cells (120). These pieces of evidence suggest that epigenetic drugs can be leveraged to enhance antitumor immunity by increasing TAA tumor antigen expression and presentation.

cGAS (cyclic GMP-AMP synthase) and STING (signaling stimulator of interferon genes) serve as cytosolic DNA sensors in both innate and adaptive immune responses (121, 122). The cytosolic DNA sensing in tumor cells by the cGAS-STING pathway triggers a signaling cascade to induce IFN production and T cell priming that arouses an antitumor immune response (123). Therefore, STING has been regarded as a master regulator of antitumor immunity (124). However, STING is frequently downregulated or silenced in BC and immune cells (125). Amplification and overexpression of MYC proto-oncogene in TNBC prevent the cGAS-STING-dependent innate immunity through binding to DNMT1 promoter and upregulating DNMT1 transcription (126). A recent study revealed that the expression of STING is epigenetically suppressed by the H3K4 demethylases, KDM5B and KDM5C. The use of KDM5 inhibitor or a combination with STING agonists in BC cells triggered a robust induction of STING expression and interferon in a cytosolic DNA-dependent manner (127). Moreover, combined use of EZH2 and HDACi induced IFI16-mediated STING activation and overcame resistance of HER2+ breast tumor to trastuzumab (128). These findings highlight the important roles of epigenetic reprogramming in modulating the cGAS-STING signaling cascade in BC.

After priming in lymph nodes, chemokine-mediated recruitment of CD8+ cytotoxic T lymphocytes (CTLs) into tumor bed is believed to be the most potent effector in the antitumor immune response. CTLs primarily eliminate cancer cells through granule exocytosis (perforin and granzymes) and death ligands, which trigger the inherent apoptotic response. Robust levels of tumor-infiltrating lymphocytes (TILs) are positively associated with favorable clinical outcomes and improved response to chemotherapy and immunotherapy in BC (129, 130). Increased expression of the C-X-C motif chemokine ligands 9 and 10 (CXCL9 and CXCL10), C-C motif chemokine ligand 5 (CCL5), and IFNγ is associated with enhanced presentation of CTLs in the TME. Our study has shown that LSD1 overexpression is negatively correlated with the level of CD8+ T cell attracting chemokines (CCL5, CXCL9, CXCL10) in TNBC (61). Re-expression of these chemokines by LSD1 depletion is associated with increased H3K4me2 levels at proximal promoter regions of chemokine genes. LSD1 inhibitor enhances CD8+ T cell migration which was blocked by concurrent treatment with siRNA or inhibitor of chemokine receptors on T cells, indicating a critical role of LSD1 in governing CD8+ lymphocyte trafficking to the tumor cluster (61). Another study showed that targeting LSD1 induces infiltration of IFNγ/TNFα-expressing CD8^+^ T cells in mice bearing 4T1 ICI-resistant tumors, which is further augmented by combined immunotherapy (131). These findings suggest the potential of targeting LSD1 in boosting antitumor immunity and overcoming resistance to immunotherapies (132). Li et al. found that JmjC demethylase JARID1B binds to LSD1/NuRD and suppresses angiogenesis and metastasis in BC cells by repressing CCL14, a chemokine-promoting the activation of immune cells (133). Chemokine (C-C motif) ligand 7 (CCL7) is a chemotactic factor and potent attractant of monocytes which plays an important role in regulating antitumor immunity and response to ICI therapy (134, 135). H3K4me3-targeting histone demethylase, Fbxl10, has been found to be recruited to CCL7 promoter and knockdown of Fbxl10 led to inverse regulation of CCL7 (136). Deng et al. showed that hypomethylation of CG sites (cg05224770 and cg07388018) was associated with upregulation of Chemokine Receptor 7 (CCR7) in BC (137). These studies suggest that targeting an aberrant chemokine network in combination with other immunotherapies may augment antitumor immunity and produce clinical benefits in patients with breast cancer.

## 4 Epigenetic modulation of immunogenic cell death in breast cancer

Immune checkpoint molecules (ICMs) expressed by innate immune cells exert inhibitory effects on adaptive immune responses. ICM family includes programmed cell death protein 1 (PD-1), cytotoxic T-lymphocyte-associated protein 4 (CTLA-4), lymphocyte-activation gene 3 (LAG-3), T cell immunoglobulin and mucin-domain containing-3 (TIM-3), and others (138). While ICMs are crucial regulators for self-tolerance and may prevent the immune system from attacking cells indiscriminately, tumor cells frequently evolve to evade immune surveillance by stimulating immune checkpoint targets and passing an “off” signal to the T cells (139). Gene expression analysis using the METABRIC microarray dataset found that the expression of ICMs is upregulated in many breast tumors, more significantly in basal-like and HER2-enriched subtypes (140). Sasidharan et al. showed that DNA hypomethylation and decreased expression of repressive histone marks H3K27me3 and H3K9me3 contribute to the upregulation of PD-1, CTLA-4, TIM-3, and LAG-3 in breast tumor tissues (141). Overexpression of PD-L1 (CD274), the ligand of PD-1, has been proven to promote immune evasion and tumor growth through enhancing T cell apoptosis in many types of cancer (142). DNA demethylation by ten-eleven translocation (TET) methylcytosine dioxygenases could lead to overexpression of IMCs in BC patients’ blood and tumor tissues (143). Darvin et al. reported that overexpression of PD-L1 in breast cancer-like stem cells was partially independent of promoter CpG methylation and more likely due to posttranslational histone modifications such as lysine tri-methylation and acetylation (144). Although PD-L1 has been correlated with poor prognosis in BC, several clinical trials have reported the positive association of PD-L1 expression with higher response rates to anti-PD-1/PD-L1 antibody therapy (145, 146). Based on these findings, some epigenetic agents could be used to improve antitumor immunity by suppressing the expression of ICMs, while others could be used in combination with ICIs to enhance tumor response to immunotherapy by upregulation of ICM expression.

Myeloid-derived suppressor cells (MDSCs), which commonly express Siglec-3/CD33 and lack HLA-DR and lineage markers, are a group of immature myeloid cells that potently suppress T cell activity and thus contribute to the immune escape of tumors (147). Results from several studies have suggested that histone modifications exert a range of effects on the immunosuppressive function and expansion of MDSCs in BC. The HDACi vorinostat reduces MDSC accumulation in the spleen, blood, and tumor bed but increases the proportion of IFN-γ- or CD8+ T cells in BALB/C mice bearing 4T1 tumors (148). Acetylation of SMAD3 by histone lysine acetyltransferase 6A (KAT6A) promotes metastasis of TNBC through the recruitment of MDSCs. Inhibition of KAT6A in combination with anti-PD-L1 therapy in TNBC-bearing mice reduced MDSC recruitment, upregulated cytokine expression (IL-6, IL-22, and TNFα), and markedly attenuated metastasis of TNBC tumors (149).

Natural Killer (NK) cells are potent effectors of the innate immune system which are best known for killing infected and cancer cells that lack MCH restriction or prior sensitization (150). Chan et al. demonstrated that DNMTi altered gene expression of inhibitory receptors of natural killer (NK) cells. Combining DNMTi with antibodies targeting NK cell inhibitory receptors, TIGIT or KLRG1, effectively reduced the metastatic potential of BC cells (151). Simultaneous implantation of mesenchymal stem cells (MSCs) overexpressing Sirt1 suppressed 4T1 breast tumor growth in mice via chemokine (CXCL10 and IFN-γ)-dependent NK cells recruitment (152). NKG2D is one of the major activating receptors of NK cells that binds to several ligands NKG2DLs. One study showed that silencing of NKG2DL inhibited the miR-17-92 cluster, especially miR-20a, promoting NK cell-mediated cytotoxicity against BC cells and inhibiting immune escape. Treatment with HDACi inhibits members of the miR-17-92 cluster leading to the induction of NKG2DL expression in multiple BC cell lines, suggesting that targeting specific miRNAs with epigenetic modifying drugs may represent a novel approach for augmenting NK cell-mediated antitumor immunity in BC (153).

Tumor-associated macrophages (TAMs) are pivotal in tumor development and anti-cancer therapy. TAMs display a high degree of cellular plasticity and exert context-dependent anti-tumor (M1-like) or pro-tumor (M2-like) functions and polarization states (154). Epigenetic mechanisms have emerged as key controllers of macrophage activation and polarization. For instance, Li et al. reported that TAMs increase DNMT1 expression in breast cancer cells via the IL-6-pSTAT3-ZEB1-DNMT1 axis and DNMT1 is required for TAM-mediated BC metastasis (155). A recent study suggested that miR-182 promotes macrophage alternative activation and drives breast tumor development. Targeting miR-182 inhibitors through delivering TAM-targeting exosomes into macrophages can effectively suppress M2 polarization and block BC progression (156). Another study showed that miR-200c promotes TNBC progression by upregulation of plasminogen activator inhibitor-2 (PAI-2) and M2 phenotype macrophage polarization (157). Hey et al. used 4T1 mouse model and single-cell gene expression data to identify a TAM-specific signature which is associated with altered cytokine production and immune factors in TNBC (158). Moreover, LSD1-CoREST complex has been found to have a role in switching macrophage polarization programs and LSD1 inhibition can prime macrophages toward an anti-tumor M1-like phenotype in TNBC (159). An improved understanding of the complex interactions between TAMs and epigenetic changes is vital to identifying novel TAM-based epigenetic therapies.

## 5 Combination of epigenetic drugs with immunotherapy in breast cancer: preclinical studies and clinical trials

Recent progress in immunotherapy represents one of the most encouraging advances in the cancer therapeutic field. However, the clinical results suggest that the general response rate for breast cancer patients treated with immune checkpoint inhibitors is around 10-20% (17, 160, 161). While TNBC is more likely to respond to immunotherapy, the overall response rate is still low. Therefore, developing rational combination therapies is a critical approach to improve the efficacy of immunotherapy in BC. The use of epigenetic drugs to prime for response to immunotherapy might lead to new strategies to boost anticancer immune responses and efficacy of immunotherapy.

### 5.1 Targeting DNA hypermethylation to improve immunotherapy

One preclinical study showed that combined treatment with anti-PD-1 and anti-CTLA-4 antibodies failed to block the growth and metastasis of 4T1 tumors, whereas cotreatment with DNMTi Azacitidine, anti-PD-1 and anti-CTLA-4 antibodies significantly improved treatment outcomes with more than 80% reduction in tumor burden (162). Another study demonstrated that 5-aza-2’-deoxycytidine significantly enhanced the tumor cell-killing effects of MAGE-As co-antigen peptide-specific CTLs and anti-CTLA-4 monoclonal antibody in BC cells (163). Recently, several studies using a combination of DNA methylation inhibitors and immunotherapy have advanced into clinical investigations for breast cancer patients. As shown in **Table 3**, one completed phase II trial has evaluated the antitumor activity of DNA hypomethylating agent azacitidine (CC-486) in combination with anti-PD-1 antibody (durvalumab) in metastatic ER-positive/HER2-negative BC and other types of cancer (NCT02811497). The published data indicated that the combination was considered safe at the dosages delivered. Although the study did not observe robust pharmacodynamic or clinical activity, lessons learned from this biomarker-rich study will inform continued drug development efforts using these agents (164). Clinical trials conducted in China have assessed the feasibility and safety of anti-PD-1 antibody (camrelizumab) alone or in combination with decitabine and/or chemotherapy in a variety of refractory malignancies including BC (NCT02961101). A completed study in patients with classic Hodgkin lymphoma (cHL) showed that DNMTi plus anti-PD-1 therapy is associated with high response rates and long-term benefits in patients with relapsed/refractory cHL who didn’t respond to PD-1 antibody therapy (165). Several other ongoing studies are investigating the efficacy of decitabine with a chemotherapy/pembrolizumab regimen for patients with locally advanced HER2-negative BC and TNBC (NCT02957968, NCT05673200). The primary goals of these trials are to determine if DNMTi could enhance the antitumor efficacy of immunotherapy and chemotherapy.

**Table 3:** Some completed and ongoing clinical trials of epigenetic drugs and immunotherapy in breast cancer (clinicaltrials.gov).

Trial Number	Phase	Condition	Intervention	Outcome Measure	Patient Number	Completion Date
NCT02811497	II	ER+/HER2- BC, CRC, OC	Azacitidine + Durvalumab	ORR, DCR, PFS, OS, AEs	28	August 2020
NCT02961101	I/II	non-Hodgkin’s lymphoma, hepatocellular carcinoma, BC, OC, lung cancer, renal-cell cancer	Anti-PD-1 Antibody Alone or in Combination with Low-dose Decitabine and/or Chemotherapy	CTCAE, RECIST, PFS, ORR, OS	250	May 2020
NCT02957968	II	BC	Doxorubicin, Paclitaxel, Cyclophosphamide, Carboplatin, Decitabine, Pembrolizumab	Infiltrating lymphocytes, AEs, pCR, CR, PD-L1, MDSC	46	February 2025
NCT05673200	I	Metastatic TNBC	Cedazuridine, Decitabine, Paclitaxel Pembrolizumab	MTD, Safety profile, Tolerability, TRR, DOR, OS. PFS	24	February 2027
NCT02453620	I	Advanced or Metastatic HER2-Negative Breast Cancer	Entinostat, Ipilimumab, Nivolumab	AEs, ratio of Teff to Treg, ORR, DCR, PFS, DOR, Duration of stable disease	57	August 2024
NCT02708680	I/II	Advanced TNBC	Atezolizumab + Entinostat	irRECIST, ORR, CBR, OS, DOR, TRR	89	March 2021
NCT03280563	I/II	HR+ or HER2- BC	Atezolizumab, Bevacizumab, Entinostat, Exemestane, Fulvestrant, Ipatasertib, Tamoxifen, Abemaciclib	RECIST, PFS, OS, AEs	138	December 2024
NCT05749575	II	Low HR Expression, HER2-negative Early Breast Cancer.	Cedardenamine +PD-1 mAb + Paclitaxel	ORR, DFS, EFS	28	August 2024
NCT05438706	II	TNBC	Chidamide (HDACi) + Camrelizumab (PD-1 mAb) and Carboplatin or Capecitabine	ORR, PFS, OS, DCR, CBR,	70	July 2024
NCT05422794	I	TNBC	ZEN003694 (BETi), Pembrolizumab (PD-1 mAb), Nab-Paclitaxel	PK, RP2D, ORR, PFS, DOR, TTOR, Biomarkers	57	December 2025

BC, breast cancer; CRC, colorectal cancer; OC, ovarian cancer; ORR, objective response rate; DCR, disease control rate; PFS, progression-free survival; OS, overall survival; AE, adverse event; CTCAE, common terminology criteria for adverse events; RECIST, response evaluation criteria in solid tumor; pCR, pathological complete response; CR, complete response; PD-L1, programmed death-ligand 1; MDSC, myeloid-derived suppressor cells; MTD, maximum tolerable dose; TRR, tumor response rate; DOR, duration of response; DCR, disease control rate; irRECIST, immune-related response evaluation criteria in solid tumors; CBR, clinical benefit rate; ORR, objective response rate; DFS, disease-free survival; EFS, event-free survival; PK, pharmacokinetics; RP2D, recommended phase 2 dose; TTOR, time to objective response.

### 5.2 Combination of HDAC inhibitor with immune checkpoint blockade in breast cancer

A preclinical study demonstrated that HDACi improved *in vivo* response to PD-1/CTLA-4 blockades in TNBC by up-regulating PD-L1/HLA-DR and down-regulating CD4^+^ Foxp3^+^ regulatory T cells (Treg) (166). Another study reported that the combination of HDACi with ICIs altered the infiltration and function of innate immune cells, allowing for a more robust adaptive immune response through suppression of MDSCs and immune-resistant breast tumors (167). Kim et al. demonstrated that the combination of entinostat with CTLA-4 and PD-1 antibodies blocked the growth and metastases of 4T1 breast tumors in mice via suppression of MDSCs (162). The combined use of HDACi as a potential booster for immune checkpoint blockade has been assessed in multiple BC clinical trials (**Table 3**). One phase I study evaluated the side effects and optimal dose of entinostat and nivolumab (anti-PD-1 mAb) when given together with ipilimumab (anti-CTLA-4) in treating patients with unresectable or metastatic HER2-negative BC (NCT02453620). The preliminary evidence of both clinical efficacy and immune modulation shows that combining entinostat with nivolumab and ipilimumab was safe and tolerable with expected rates of immune-related adverse events (168). This drug combination is being evaluated further in an expansion cohort of HER2-postive BC patients. However, another phase II, randomized, placebo-controlled, double-blinded, multicenter study of atezolizumab (anti-PD-L1 mAb) with entinostat in patients with advanced TNBC did not find prolonged median PFS compared with entinostat alone and the combination resulted in greater toxicity (NCT02708680). One ongoing study is evaluating the efficacy of several immunotherapy-based combination treatments, including entinostat in participants with inoperable locally advanced or metastatic BC who have progressed during or following treatment with a cyclin-dependent kinase (CDK) 4/6 inhibitor (palbociclib, ribociclib, or abemaciclib) (NCT03280563). Other ongoing clinical trials are testing if Chidamide, an HDACi developed in China, could sensitize breast tumors to the antitumor effects of PD-1 antibody and chemotherapeutic agents such as paclitaxel, carboplatin, or capecitabine in relapsed/metastatic HER2-negative or TNBC (NCT05749575, NCT05438706).

### 5.3 Emerging new epigenetic targets for breast cancer immunotherapy

We have demonstrated that inhibition of LSD1 by RNAi or small molecule inhibitors reactivates T-cell attracting chemokines which may in turn stimulate CD8+ T cell infiltration and sensitize poorly immunogenic TNBC tumors to PD-1 blockade (61). This combination approach also augmented the ratio of CD8+/CD4+ T cells in lymph tissues adjacent to tumor sites at mouse mammary glands, which is considered an important marker of immunological defense against tumor cell dissemination (61). These findings support the promise of LSD1 inhibition as an effective approach to overcoming resistance to ICIs in BC treatment. One ongoing Phase I/II study sponsored by the University of Washington is evaluating the effect of an orally active LSD1 inhibitor, bomedemstat (IMG-7289), and immunotherapy in patients with newly diagnosed extensive stage small cell lung cancer (Es-SCLC) (NCT05191797). It is anticipated that the outcome of this clinical study will provide beneficial information for trials using LSD1 inhibitors in other types of cancer.

BET inhibitors (BETi) have emerged as a novel class of epigenetic drugs that acts as an anti-cancer agent by blocking VEGF production and down-regulating MYC expression in different types of cancer, including BC (169). A number of inhibitors targeting BET have been reported, and many preclinical studies and clinical trials are under way to examine the anti-cancer efficacy of BETi alone or in combination with other therapeutic agents. A recent study has indicated that BRD4 inhibition suppressed PD-L1 expression and changed the proportions of T lymphocyte subsets in mice bearing TNBC tumors (170). Lai et al. developed a mathematical model to show that the BETi and CTLA-4 inhibitor are positively correlated in tumor inhibition and sustain cytotoxic T cell function in BC (171). Furthermore, BET inhibition suppresses the PD-1/PD-L1 axis, improves tumor cell-specific T cell cytotoxic function, and overcomes tumor-mediated T cell exhaustion in TNBC (172). NCI is sponsoring a phase Ib trial using a BET inhibitor, ZEN003694, in combination with pembrolizumab (PD-1 mAb) and nab-paclitaxel for patients with locally advanced or metastatic TNBC (NCT05422794). The outcomes of these trials will provide useful information to address whether targeting dysregulated histone marks would enhance the efficacy of ICIs for breast cancer patients.

## 6 Conclusions and future prospects

Over the past decades, the rapid development in understanding of epigenetic alterations in breast cancer has facilitated the development of many new diagnostic and treatment tools for this disease. The great potential for epigenetic therapy lies in the knowledge that epigenetic changes can be reversed, allowing restoration of function of aberrantly affected genes in tumor cells. Many drugs targeting dysregulated epigenetic regulators have entered clinical use in the treatment of hematological cancers. The FDA has approved EZH2 inhibitor Tazemetostat for epithelioid sarcoma, the first approval of an epigenetic drug for solid tumor. Increasing and compelling evidence suggests that epigenetic therapy has the potential to convert an immune-repressive (cold) breast tumor into an immune-permissive (hot) one. Recent studies have identified many new roles for epigenetic alterations in promoting immune escape that may represent an opportunity to identify novel therapies for immunotherapy-refractory breast cancer. Given the fact that epigenetic alterations exert effect on both breast tumor cells and immune cells, it is hoped that the combination of epigenetic drugs with immunotherapies can consolidate the antitumor immune response, reprogram the immune suppressive microenvironment, and improve therapeutic efficacy. However, many challenges remain to be addressed and solved in the future. The ultimate goal is to mitigate the off-target effects of epi-drugs and explore more effective site-specific drug delivery approaches. A better understanding of the underlying mechanisms of epigenetic regulation in BC is key for the development of more specific epigenetic-based therapy. In recent years, the progress in epigenome-wide studies has led to the discovery of many previously unknown epigenetic modifiers, especially the histone modifying enzymes. These newly identified epigenetic targets have potential to serve as novel diagnostic and prognostic biomarkers for breast cancer. However, the similarity among enzyme families may elicit cross-reactivity of substrates and inhibitors and impair the specificity of epigenetic drugs. A better insight into the chromatin-context specificity of histone modifying enzymes would facilitate the development of more effective and selective inhibitors. As breast cancer is a heterogeneous disease with multiple subtypes and distinct biological features, we need to further elucidate the diversity of epigenetic regulation in the tumor immune environment. Finally, the epigenetic landscape in immunotherapy-refractory breast tumor populations should be further defined and characterized to identify unique biomarkers in order to optimize the combination of immunotherapy and epigenetic-based therapy with the long-term goal to expand the responder population and improve personalized precision medicine.

## Author contributions

JY: Conceptualization, Writing – original draft. TG: Writing – review & editing. NC: Writing – review & editing. ND: Writing – review & editing, Funding acquisition. YH: Funding acquisition, Writing – review & editing, Conceptualization, Data curation, Resources, Software, Supervision, Visualization, Writing – original draft.
